# That Compositor Again

**Published:** 1897-12

**Authors:** 


THAT COMPOSITOR AGAIN.—In the November number of this Journal, notwithstanding the proof was distinctly marked for correction, the chump at the printing office made me say "not receive the attentions of no man." Now isn't that enough to make even so pious a man as yours truly say some cuss words? We can only throw ourself on the mercy of our readers and beg that, in the light of a few antecedent evidences of the possession of some intelligence on our part, they will not conclude that the writer is "just a plum idiot;" that's all.

